# Social prescribing for suicide prevention: a rapid review

**DOI:** 10.3389/fpubh.2024.1396614

**Published:** 2024-07-05

**Authors:** Sarah Dash, Stella McNamara, Maximilian de Courten, Rosemary Calder

**Affiliations:** Australian Health Policy Collaboration, Victoria University, Melbourne, VIC, Australia

**Keywords:** suicide, social prescribing, efficacy, prevention, review, evidence

## Abstract

This rapid review delves into the realm of social prescribing as a novel approach to suicide prevention by addressing the social determinants of health. Through an exploration of various databases including MEDLINE, PsychInfo, WILEY, and Sage, a total of 3,063 articles were initially identified as potentially relevant to the research. Following a meticulous screening process, 13 articles were included in the final review, shedding light on the potential effectiveness and impact of social prescribing interventions on suicide prevention. Key findings indicate the need for additional monitoring and support for individuals at risk of suicide, emphasising warm referrals and sustained connections after referral to enhance the efficacy of social prescribing models. The review also highlights the importance of social capital and trust among vulnerable populations, underscoring the significance of community-based referrals in suicide prevention initiatives. Overall, this review identifies the potential of social prescribing as a valuable tool in mitigating suicide risk factors and promoting mental health and wellbeing in diverse populations.

## 1 Introduction

Social prescribing involves the referral of individuals to non-clinical care to address or prevent adverse effects of the social, environmental and economic factors that are inextricably linked with health and wellbeing. These are commonly referred to as the social determinants of health.

Social prescribing recognises that improving health or managing health conditions for individuals can require more than clinical care and that health professionals do not necessarily have the expertise, resources or time to address these needs (1–5). This additional form of prescribing enables health professionals to refer patients with social or practical needs that contribute or potentially will contribute to poor health, to a local community provider of non-clinical services (1, 3–6). This enables a wider range of options for care and management to be provided at the primary care level.

Social prescribing models have been developed internationally in the United Kingdom, Europe, United States, Canada, New Zealand, Scandinavia, Asia and Australia. In Australia, there are currently a growing number of practice or area-based programmes in several states. A trial of social prescribing to support mental health, particularly for older people, has been initiated in the state of Victoria following recommendations by a Royal Commission into Victoria’s Mental Health System (7).

Most recently, the Commonwealth and the state of Queensland have announced a new trial of Distress Brief Support, a two-week programme to support people experiencing psychological distress, offer practical solutions to manage that distress and identify additional services to aid longer-term recovery (8). The trial will be undertaken in two sites in Queensland and is to provide access to non-clinical support for people who are experiencing distress and who may be at heightened risk of suicide.

Social determinants of health, chronic illness, loneliness, mental health and wellbeing are all inextricably linked to suicide risk (9). Suicide prevention or management of suicidal distress is not explicitly targeted by existing social prescribing models in Australia. However, the current trials, and particularly the recently announced Queensland trials, of social prescribing directly inform suicide prevention given the shared underpinnings between chronic disease, social isolation, and suicide risk (e.g., social determinants of health, social capital, etc.).

Social prescribing, as an adjunct to clinical care and a resource for health professionals, particularly in primary health care, can form a bridge between the clinical care setting and the community sector to connect people to practical help to address social factors contributing to risks for suicide and influence wellbeing.

## 2 The evidence for social prescribing in prevention

The broader social prescribing literature supports the benefits of social prescribing for health and wellbeing, as well as the general acceptability of social prescribing for both patients and clinicians.

Suicide risk is understood to be a complex combination of biological, psychological, clinical, environmental and social factors (10). Although broader social prescribing literature has not been developed through an explicit suicide prevention lens, this paper assesses relevant evidence that the social and health benefits of social prescribing more widely are also relevant and applicable to suicide risk.

There is a significant overlap between risk factors for suicide and broader health concerns (10). For example, common psycho-social risk factors; certain physical illnesses and socio-economic factors; and some mental health conditions (11); as well as overlap in population-level approaches using risk factors.

Social prescribing has been found to be effective in reducing depression and anxiety (12). While positive results about social prescribing continue to accumulate, there remain weaknesses in the available research evidence as many studies are small-scale, do not have a control group, and focus on progress rather than outcomes, or relate to individual interventions rather than the social prescribing model. This makes the call for rigorous evaluation protocols even more pertinent (13).

Social prescribing may be able to play a role in suicide prevention by providing patients with access to community-based support services that can help address the underlying social determinants of health that contribute to suicide risk (12). Social prescribing can help address social isolation and loneliness, which are known risk factors for suicide (14).

Additionally, social prescribing models for broader health and wellbeing are likely to share many characteristics with models for suicide prevention. Though this literature is not focused explicitly on suicide prevention, this evidence is relevant in considering the efficacy and feasibility of suicide prevention models.

## 3 Social prescribing for suicide prevention: a rapid review

To build on the broader social prescribing literature and to examine the evidence specific to social prescribing for suicide prevention, a rapid review of the literature was conducted.

### 3.1 Methods

This review was guided by interim guidance on rapid reviews from the Cochrane Review Methods Group (15). A rapid review expedites the process of conducting a traditional systematic review by streamlining or omitting certain steps to produce evidence in a resource-efficient way. Complete details of the rapid review methodology can be viewed elsewhere (15).

Databases were selected to include specialised databases relevant to suicide prevention and/or social prescribing and were limited for the purposes of this rapid review. A search of databases Medline EBSCOhost, PsychInfo, Wiley and Sage was conducted. Search terms included social prescribing, suicide and efficacy with their related terms. A supplemental search was conducted of authors’ existing libraries, including grey literature. The search strategy was peer reviewed by all authors and details are included in Appendix 1.

All articles were initially reviewed by title and abstract, and all remaining articles underwent full-text review. Based on the aims, timeline and resources of this project (to inform rapid decision-making), a single author conducted the review with consultation of co-authors where required. A single author extracted data and synthesised the evidence.

Publications were included if they (i) addressed social prescribing for suicide or suicide risk factors (ii) included an evaluation component (iii) included referral outside of the medical system and (iv) were published in English. Publications were excluded if they (i) did not include community referral (e.g., referred only to an emergency department or helpline), (ii) focused only on a standalone suicide prevention intervention (e.g., gatekeeping), (iii) did not include evaluation component, (iv) were not explicitly about suicide (v) were not about social prescribing (vi) or a full text could not be sourced.

**Figure 1:**
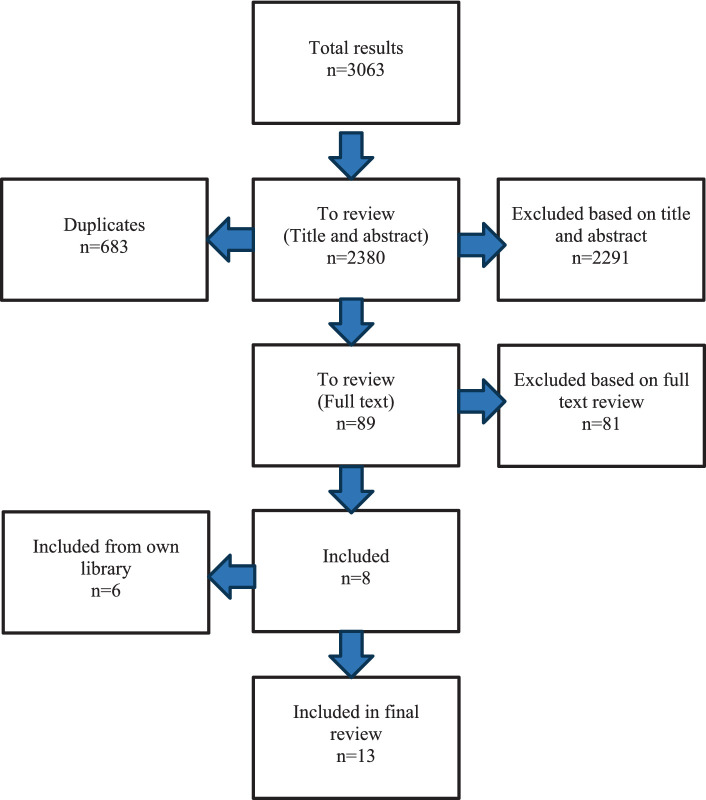
Summary of rapid review process.

A total of 3,063 publications were identified from all databases, of which 683 were duplicates. Publications were evaluated for eligibility against inclusion and exclusion criteria. After review of 2,380 titles and abstracts, 89 publications were selected for full-text review. After full-text review of the 89, a total of 8 publications met the inclusion criteria and were included in this rapid review. Reasons for exclusions included (i) did not include community referral (e.g., referred only to an emergency department or helpline) *n* = 27 (ii) focused only on a standalone suicide prevention intervention (e.g., gatekeeping) *n* = 27, (iii) did not include evaluation component *n* = 5 (iv) were not explicitly about suicide *n* = 9 (v) were not about social prescribing *n* = 11 (vi) or a full text could not be sourced *n* = 4. An additional 6 publications were identified from the authors’ libraries and were also included in the review. A summary of the review process is outlined in Appendix 1 (Figure 1).

### 3.2 Results

#### 3.2.1 Social prescribing: addressing risk factors of suicide

Most of the included literature examined social prescribing models relevant to suicide prevention through suicide risk factors, such as loneliness, social isolation or mental health concerns (Table 1). Two recent systematic reviews evaluated the literature on the impact of social prescribing on social risk factors for suicide. Reinhardt et al. (16) assessed the impact of social prescribing programmes on loneliness. A total of nine studies met inclusion criteria, all of which, as reported by Reinhardt et al., described overall positive impacts of social prescribing programmes. Three of the studies reported a reduction in service use (e.g., GPs, social worker) and one demonstrated that belongingness reduces both loneliness and healthcare use.

**Table 1:** Social prescribing publications addressing risk factors of suicide.

Study ID	Study design and setting	Participants or sources	Outcomes assessed	Conclusion
Vivodic et al. (2021)	Systematic review	51 studies included	loneliness, social isolation, connectedness and wellbeing	Individuals: evidence of change in loneliness and wellbeing is clearer than social isolation and connectedness System: evidence of change in health care, less so social care Community: Changes most evident in community resources
Wolk et al. (2021)	Implementation evaluation using RE-AIM framework of primary care referrals	Primary care clinicians in 8 practices referred 6,124 unique patients in 12 months	Mild to moderate depression, serious mental illnesses, acute suicidal ideation	Remission of symptoms in approximately a third of participants; programme was viewed favourably by stakeholders
Farr et al. (2022)	Qualitative interviews with service providers and users of Hope service, Bristol, England	16 service users men at risk of suicide (aged 30–64), six Hope staff, two specialist money advice workers funded to work for Hope and two NHS referral staff	Acceptability from a service user, staff and referrer perspective	Service provided essential support for men that would otherwise fall through the cracks. The service built trust, specialist advice tackled complex problems and supported men in gaining a sense of control. Specialist counselling for histories of abuse was hard to access.
Scott et al. (2020)	Interviews and focus groups within English Ambulance Service	Clinical and non-clinical staff from an English Ambulance Service	Awareness of social prescribing, identify patient cohorts that would benefit from social prescribing and explore barriers and enablers	Limited awareness and knowledge of social prescribing, but the benefits well recognised when it was explained. Considered most relevant for mental health, loneliness, social isolation, older people and frequent service users. Determinants of SP identified at micro (acceptability), meso (referral pathways) and macro (funding) levels.
Dayson et al. (2020)	Qualitative case study within one mental health social prescribing service with three nested case studies of social prescribing providers	20 semi-structured interviews with commissioners, providers, patients in Rotherdam, England	Wellbeing outcomes and important characteristics of a SP referral in producing wellbeing outcomes	SP makes a positive contribution to emotional, psychological and social wellbeing of patients of service. Key enablers were supportive discharge pathways with sustained community engagement activities.
Rhodes and Bell (2021)	Semi-structured qualitative interviews with social prescribing link workers	Nine SP link workers from five NHS and voluntary sector organisations, London, England	Explore the role of social prescribing link workers and identify training and support needs.	Three key themes of required support: (1) defining and promoting their role (2) supporting clients with complex needs (3) coping with the emotional demands of their role. Most felt initial training did not prepare them for all demands of role.

Similarly, Vivodic et al. (17) conducted a systematic review of the impact of social prescribing on loneliness, social isolation, connectedness and wellbeing. They examined a total of 51 studies of adults aged 18 or older. When looking at individual outcomes, the authors identified that findings were clearer in relation to loneliness and wellbeing compared to social isolation and connectedness. System-level findings of this review include reductions in health care usage (e.g., emergency department visits, healthcare appointments). Importantly, Vivodic et al. identify that few studies made clear causal links between positive outcomes and the social prescribing model. Authors identified barriers to effective programme delivery (e.g., patient accessibility, funding). However, there are few descriptions of key components of social prescribing models. Both systematic reviews identify significant variation in measuring outcomes and identifying pathways of impact and call for improved evidence on how social prescribing works and how best to define its impact.

One study tested a model of collaborative care for referred patients with unmet mental health needs. Wolk et al. (18) developed and implemented the US-based Penn Integrated Care programme; a new model of collaborative care that includes a triage and referral management system based on a resource centre that also provided support for individuals to be referred to appropriate community health services and resources. The programme was trialled by primary care clinicians in 8 practices. Patients with specific conditions, ranging from mild to moderate depression, serious mental illnesses, to acute suicidal ideation, were referred to community programmes based on clinical assessment, their preferences, insurance coverage and information from the primary care clinician. The centre then assisted with scheduling an appointment and followed up to ensure the individuals attended and engaged with care. If not, the centre linked them to other services. Mental health professionals were available for ‘warm’ referrals when patients were in crisis, however, most referrals were conducted electronically. Where appropriate, patients were referred to community-based programmes, psychiatrists or specialists. In 12 months, over 6,000 patients were referred, primarily to collaborative care (26%) or specialty mental health care with active referral management (70%). Of the over 6,000 referred to the programme, approximately 3,500 were provided with resources and referrals, the majority of whom (approx. 2,500) were provided community referrals. Patients enrolled in collaborative care had an average of 7 encounters over an average of 78 days. Remission of symptoms was obtained in approximately a third of participants and the programme was viewed favourably by stakeholders. Although this model does not examine all types of community referral (e.g., addressing social determinants of health), it demonstrates some efficacy of community referral in addressing suicide risk factors and active suicidal ideation.

More specifically, four studies describe qualitative findings of social prescribing models for suicide risk factors. Farr et al. (19) described the Hope service, a programme developed for men at risk of suicide (aged 30–64) to provide psychosocial and practical advice in relation to money, employment and housing that is based in Bristol, England. A previous pilot randomised trial found it to be feasible and acceptable (20). This study aimed to evaluate the acceptability from a service user, staff and referrer perspective and to understand which factors of the programme influence its impact. The programme used a project team member who delivered up to 8 face to face sessions within this intervention to connect each user to other agencies. While not described as such, the intervention was that of a ‘link worker’, a role now well established in social prescribing services that provides the link between the referring health or other professional and the community support sector relevant to a person’s identified or assessed needs. The evaluation research identified key elements of the programme including creating a safe space, building trust and specialist advice on psychosocial problems. The researchers also found that suicide ideation in men was closely linked to life crises. Addressing social factors improved a sense of control, which supported mental health. Men may also have felt less threatened by Hope project workers than those in mainstream health services. The authors noted limitations of interviewing those who were well engaged with the programme but reported that the service overall was considered useful and important.

Scott et al. (21) published a qualitative study exploring the potential for a social prescribing model within pre-hospital emergency and urgent care in England. They specifically examined groups that might benefit from this model, including those with suicidal risk factors. They conducted interviews (*n* = 15) and a focus group (*n* = 3) with clinical and non-clinical staff from an English Ambulance Service covering emergency and non-emergency calls. They wanted to determine awareness of social prescribing, identify patient cohorts that would benefit from social prescribing and explore barriers and enablers. Participants had varying levels of awareness of social prescribing. Key groups identified as suitable cohorts were patients with mental health conditions, lonely and/or socially isolated groups and older people and frequent callers. They identified key criteria for implementing a social prescribing model, including patient and staff acceptability of the model, knowledge of services, available triage pathways, funding and commissioning and equitable access across areas. At a micro level, they identified the importance of the acceptability of social prescribing; at a meso level, the importance of triage and referral pathways and at a macro level, that social and health infrastructure is essential.

Dayson et al. (22) described a social prescribing model within the NHS in England, which currently operates in primary but not secondary care. This existing primary-care based model was unable to handle referrals from community mental health services, so a second model was needed. The service helps patients tailor packages of support and enables them to participate in peer-led community events. Community mental health centres and link workers work together for 10 weeks to ensure patients are engaged with community-based activities and community mental health centres remain involved for up to 6 months. The authors conducted 20 semi-structured interviews with a mix of commissioners, service providers and patients accessing social prescribing. Patients reported improvements in quality of life and identified that social prescribing activities brought a sense of purpose, particularly that they enabled integration for previously isolated patients. The supportive transition model was very important. Not all participants engaged and/or could be discharged, so social prescribing may not be effective for everyone.

Lastly, Rhodes and Bell (23) conducted semi-structured interviews with nine social prescribing link workers across five organisations in London, exploring the role of link workers and examining training and support needs. While these link workers were not operating in an explicit suicide prevention model, they encountered suicide risk within their role. Key support needs included defining and promoting their link worker role, coping with the emotional challenges of the role and managing clients with complex needs. Most link workers felt their training was not adequate for the most challenging parts of their role, which often included suicide risk.

Overall, the literature on social prescribing for suicide risk factors indicates some positive impacts on suicide risk factors such as loneliness, belonging, social connectedness and sense of purpose. However, there are limitations to drawing causal links between social prescribing models and these outcomes, and further research is warranted. Although these models were generally considered acceptable by patients and staff, several authors outline infrastructure-associated barriers to implementing or scaling up these programmes.

#### 3.2.2 Social prescribing for suicide bereavement and prevention

Three studies included in the rapid review examined social prescribing models that included suicide (Table 2). Importantly, two of these studies focused on suicide bereavement and one included those with reduced social support, an important suicide risk factor.

**Table 2:** Social prescribing for suicide bereavement and prevention.

Galway et al. (2019)	Contextual analysis, Consultation, stakeholder engagement, co-design	Initial roundtable (*n* = 10) and face-to-face meetings with providers and commissioners (*n* = 16 organisations), co-design workshops (*n* = 29; 19 females and 10 males), Belfast	Acceptability of adapting digital social prescribing for suicide bereavement support	There was a consensus that DSP could potentially improve access, reach, and monitoring of care and support. Stakeholders also recognised the potential for DSP to contribute substantially to the evidence base for postvention support.
Hill et al. (2022)	Retrospective cross-sectional mixed methods approach	Suspected suicide data from 1 Jan 2019 to 31 March 2021 linked to data from service provider in Western Australia	(1) Identify the reach of the PCN model, (2) describe the type of support provided to people bereaved by a suspected suicide and (3) identify the perceived effectiveness of the PCN model from the perspective of police, postvention stakeholders and individuals bereaved by suicide.	80 suspected suicides during study period, active outreach to 347 bereaved individuals via PCN model. Under half (*n* = 164) accepted further support. Police, key stakeholders and those with lived experience deemed the model to be effective at linking to community, support and in preventing suicide.
Petrakis and Joubert (2013)	Model of assertive brief psychotherapeutic intervention and facilitated linkage to community services utilised in a prospective cohort study of emergency department suicide attempt aftercare.	65 patients, assessed psychosocial domains at initial presentation, 4-weeks, 3-months, and 6-months in Melbourne, Australia	The Manchester Short Assessment of Quality of Life (MANSA)	Significant improvements in domains of work, finance, leisure, social life, living situation, personal safety and health by 3 months. There were highly significant correlations between psychosocial improvements and improved depression scores.

Studies by Galway et al. (24) and Hill et al. (25) both tested social prescribing for suicide bereavement support. Galway et al. tested the acceptability of adapting digital social prescribing for suicide bereavement support based in North Ireland. There was a consensus that digital social prescribing could potentially improve access, reach and monitoring of care and support. However, the stigma of care, reluctance to access support, matching types of support to needs and some limitations of digital resources (e.g., rural areas, limited internet) were noted.

Exploring a more traditional social prescribing model, Hill et al. examined a Primary Care Navigator model for people bereaved by suicide. This took place in Western Australia, and bereaved individuals were referred by police into the model. The Primary Care Navigator assessed the needs of the person(s) referred and connected them with other community services (e.g., meals, housing, sporting clubs) as needed. Over 15 months there were 90 suspected suicides and this model reached 347 bereaved individuals, just under half of whom accepted further support. While bereavement information and clinical support were the most prevalent, individuals also accessed financial assistance, meals, housing assistance, and referrals to community services (11–16%). This model was perceived to be effective by police, stakeholders and people with lived experience of a suspected suicide.

Lastly, Petrakis and Joubert (26) evaluated an intervention that, among other objectives, focused on facilitated community linkage responding to impaired social support. This was monitored through the number of referrals and subsequent engagement with existing community resources. Although the details of linkage pathways are not clearly outlined, the authors make practice recommendations about improving the interface between acute care and community care. Without monitoring, patients often do not follow up on referrals as advised. Particularly among patients with depression, monitoring and support is required for referral uptake and retention.

These studies highlight that literature explicitly taking a prevention-based approach to suicide prevention through social prescribing is limited. Importantly, engagement and effectiveness data of suicide bereavement may not readily translate to suicide prevention (e.g., people may be more likely to engage with social prescribing as an early intervention, rather than amidst a crisis). Given the unique needs and challenges of a suicide prevention social prescribing service, additional research is required.

#### 3.2.3 Social prescribing pilots in Australia

There are several social prescribing programmes targeting risk factors associated with suicide that have been trialled and evaluated in Australia. Although other programmes may be ongoing, just three programmes were retrieved during rapid review and met inclusion criteria (Table 3).

**Table 3:** Social prescribing pilots in Australia.

Aggar et al. (2021)	Evaluation of a social prescribing pilot using an exploratory, quantitative, longitudinal design	13 adults who were assessed at baseline and six-month follow-up	Self-perceived quality of life, welfare needs, health status, loneliness, social participation, and economic participation	Significant improvements in quality of life and health status
Doran et al. (2021)	Evaluation of the MATES case management database	3,759 individuals collected over the period 2010–2018, and exit survey undertaken with 14 clients in 2019	To quantify service demand, and to examine the demographic, occupational profile, presenting issues, referral pathways, and perceived benefit of case management among individuals who used this service.	Demand for case management through MATES has increased significantly. Clients felt that their needs/ concerns were appropriately addressed. Most common presenting issues were relationship, work, and family problems, suicide, and mental health concerns. Causes of distress span a range of psychosocial issues, beyond mental health. Offers an approach that redirects people towards services equipped to meet their needs and away from over-run emergency departments.
Gullstrup et al. (2023)	This systematic review of the available evidence for the effectiveness of the MATES program	12 peer-reviewed articles published between January 2010 and February 2023 containing primary data of evaluations of MATES.	Effectiveness of the MATES program	Evidence of the effectiveness of the MATES programme in improving mental health and suicide prevention literacy, helping intentions and reducing stigma. Workers stated supervisors were least trusted resource for mental health and suicide concerns Favourable results to reduced suicide rates in construction industry The evidence base for MATES is limited with few controlled evaluations and no experimental studies to date
Dingle et al. (2023)	Trial comparing General Practitioner treatment-as-usual (TAU) with TAU combined with Social Prescribing (SP)	114 individuals experiencing loneliness in Queensland were non-randomly assigned to one of two conditions – social prescribing and treatment as usual	Assessed at baseline and 8-weeks on primary outcomes (loneliness, well-being, health service use in past 2 months) and secondary outcomes (social anxiety, psychological distress, social trust).	High retention (79.4%) in social prescribing group. Time x group interaction for loneliness and social trust. Improvement only in social prescribing group at 8 weeks. Social prescribing group reported improvements (small-to moderate effect sizes) on all other outcomes (loneliness, wellbeing, psychological distress, social anxiety) however interaction effects did not reach significance.

In 2021, Aggar et al. (27) published Social Prescribing for Individuals Living with Mental Illness in an Australian Community Setting: A Pilot Study. The authors describe this as Australia’s first social prescribing pilot programme (Plus Social) for individuals with mental illness (mood and psychotic spectrum disorders), and the programme was implemented in Sydney in 2016/2017. This study provides an evaluation of that programme.

A total of 13 individuals participated and were assessed at baseline and six-month follow-up; results indicate significant improvements in quality of life and health status. Participants were referred by a GP into the programme and were assessed by a mental health social worker (link worker) who referred onwards (e.g., NSW Health House and Accommodation Support Initiative) as needed. All participants also attended weekly arts and crafts classes. The results indicate that participants who completed the programme experienced significant improvements in psychological and physical quality of life, health satisfaction, and self-perceived health status. Importantly, the results show no significant differences in social participation and self-rated loneliness, although scores suggest participants experienced less loneliness through the duration of the study.

Results of an Australian-based workplace suicide prevention and early intervention programme called MATES in Construction were also published in 2021 (28). The paper evaluates service demand, demographic and occupational profile of users, reasons for access, referral pathways and perceived benefits.

MATES in Construction was developed by the Building Employees Redundancy Trust in 2008 to prevent suicide in the construction industry. The programme offers mental health training, non-clinical case management, an outreach service and a 24-h support service to employees. Previous evaluations of the programme demonstrated its validity, effectiveness in shifting beliefs around suicide, improved suicide prevention literacy and increased intentions to seek help for themselves, as well as significant economic return on investment.

The programme uses a case management approach, though MATES case managers do not provide mental health care to clients. They use a brokerage model where case managers endeavour to help clients identify services and broker supportive services over a short contact period. This model assumes the individual will voluntarily access services when they know what is available and how to access them. The focus is less on direct service to the client, and a focus on assessing needs, planning a service strategy, connecting and following up with clients. Clients are most commonly referred to Employee Assistance Programmes, followed by mental health, counselling or wellbeing services and a small proportion were referred to medical services. The findings of this evaluation indicate that clients felt their needs were addressed. Results also confirm that presenting issues include a range of psychosocial concerns.

Recently, Gullstrup et al. (29) conducted a systematic review on the effectiveness of the MATES in Construction program. The review included 12 peer-reviewed articles published between 2010 and 2023. The review identified evidence to support the effectiveness of the MATES programme in improving mental health and suicide literacy among participants, helping intentions, and reducing stigma surrounding mental health. These results were positive in relation to reduced suicide risk in the construction industry, but few studies were well-controlled and there were no experimental studies. Therefore, more research is required to understand the causal relationship between MATES and suicide risk.

Lastly, a pre-print of a paper published by Dingle et al. (30) provides a controlled evaluation of 8-week outcomes of a social prescribing project addressing loneliness in adults in Queensland. The trial compared (1) treatment as usual only with (2) treatment as usual plus social prescribing among adults experiencing loneliness. A total of 114 participants were assigned to the two groups and were tested at baseline and at 8-weeks on a range of wellbeing metrics including loneliness and wellbeing. The findings showed a time with condition interaction with only the social prescribing group showing improvements over 8 weeks. Although there were small-moderate improvements on other measures (e.g., psychological distress, loneliness, wellbeing, social anxiety) among the social prescribing group, these were not significant.

Participants were recruited from five GP clinics and/or community centres and allocation to treatment group was not randomised. Participants who were allocated to treatment as usual either declined the social prescribing group or referral wasn’t feasible, or their GP did not’ consider referral necessary. Importantly, there were some baseline differences between participants who opted to participate in social prescribing—e.g., the social prescribing group reported being more challenged. Over the first 8 weeks, loneliness decreased in social prescribing patients but increased in treatment as usual patients. Overall, compared to treatment as usual, there were significant effects on loneliness and trust among the social prescribing group.

These findings suggest that social prescribing models that address suicide prevention and/or suicide risk factors are only just beginning to emerge in the literature. In alignment with broader social prescribing evidence, these studies demonstrate that social prescribing models were generally effective at addressing needs and reducing risk factors such as loneliness. Generally, these studies did not provide substantial detail on the development of their models or the logistics of referral, indicating the importance of consulting with experts embedded in this work.

## 4 Discussion

The literature review on social prescribing for suicide prevention has unveiled crucial insights that can inform the development of effective intervention models. Key findings emphasise the necessity for tailored monitoring and support mechanisms for individuals at risk of suicide, highlighting the importance of ensuring follow-through in referrals. Moreover, the significance of warm referrals and sustained relationships in social prescribing initiatives for suicide prevention has been underscored, particularly in populations with lower levels of social capital and trust. These findings collectively advocate for a holistic and community-centred approach to suicide prevention through social prescribing.

Despite the valuable insights gained from the rapid review, several limitations warrant consideration. The limited number of studies included in the review may restrict the generalizability of findings to broader populations. Additionally, the focus on English-language publications may have introduced language bias, potentially overlooking valuable contributions from non-English sources. Furthermore, the rapid nature of the review process may have constrained the depth of analysis and synthesis of findings, necessitating further in-depth investigations to validate the efficacy of social prescribing in diverse contexts.

Several key considerations emerged from the literature that should be considered in developing a social prescribing model for suicide prevention, including:

- Additional monitoring and support of referrals may be required among those at suicide risk to support follow-through (26).
- Given lower levels of social capital and social trust among those at risk for suicide (31, 32), warm referrals and ongoing connections/relationships are important in social prescribing models for suicide prevention.
- Using a system-level approach, Scott et al. (21) note three key aspects of intervention:
  - at a micro level, the acceptability of social prescribing is needed;
  - at a meso level, triage and referral pathways are necessary; and
  - at a macro level, social and health infrastructure is required.
- Those at risk of suicide are considered particularly complex and challenging for link workers (23) and additional training and resourcing may be required.
- Digital services are being explored for suicide bereavement support, but their application is currently limited to digital outcomes-based reporting to improve the capacity for measuring the effectiveness of interventions.

In conclusion, the findings from this rapid review underscore the potential of social prescribing as a promising avenue for suicide prevention by addressing social determinants of health and fostering community connections. Moving forward, it is imperative to enhance the evidence base through rigorous evaluation of social prescribing interventions tailored to individuals at risk of suicide. By integrating warm referrals, ongoing support, and community engagement into social prescribing models, healthcare systems can better equip themselves to prevent suicide and promote mental wellbeing effectively. This review sets the stage for future research and implementation efforts aimed at harnessing the power of social prescribing in mitigating suicide risk factors and enhancing overall health outcomes.

## Author contributions

SD: Data curation, Writing – original draft, Writing – review & editing. SM: Data curation, Project administration, Writing – review & editing. MC: Methodology, Supervision, Writing – review & editing. RC: Conceptualization, Data curation, Funding acquisition, Methodology, Resources, Supervision, Writing – review & editing.

## Appendix 1: Rapid review methods

**MEDLINE.**

((“social prescri*” OR referral OR pathway OR linkage)) AND suicid* AND ((efficacy OR effectiveness OR impact OR benefits OR outcomes OR reduction))

English only.

Results: 1691.

**PsychInfo.**

((“social prescri*” OR referral OR pathway OR linkage)) AND suicid* AND ((efficacy OR effectiveness OR impact OR benefits OR outcomes OR reduction))

(All Text)

English only.

Results: 1217.

**WILEY.**

““social prescribing” OR “social prescription”“and “suicid*” and “efficacy OR effectiveness OR impact OR benefits OR outcomes OR reduction.”

(Anywhere)

Results: 85.

**Sage.**

“social prescribing” OR “social prescription” AND suicid*.

*Note that including ‘outcome’ search led to 0 results in this database.

Results: 59.

**Inclusion/Exclusion Criteria:**

Papers were included if they focused on social prescribing for suicide or suicide prevention risk factors, had some component of quantitative or qualitative evaluation, and included referrals outside of the medical system. Papers were excluded if they were in a language other than English, focused only on a single intervention (e.g., gatekeeping trials) or did not include community referrals.
